# Relation of postoperative serum S100A12 levels to delirium and cognitive dysfunction occurring after hip fracture surgery in elderly patients

**DOI:** 10.1002/brb3.1176

**Published:** 2018-12-11

**Authors:** Qing‐Hua Li, Liang Yu, Zheng‐Wei Yu, Xiao‐Liang Fan, Wang‐Xiang Yao, Cheng Ji, Fang Deng, Xian‐Zhe Luo, Jian‐Liang Sun

**Affiliations:** ^1^ Department of Anesthesia and Pain, The Affiliated Hangzhou First People’s Hospital Zhejiang University School of Medicine Hangzhou China; ^2^ Department of Orthopedics, The Affiliated Hangzhou First People’s Hospital Zhejiang University School of Medicine Hangzhou China

**Keywords:** cognitive dysfunction, delirium, elderly, hip fracture, postoperative, S100A12

## Abstract

**Objective:**

Brain injury is implicated in pathogenesis of postoperative delirium (POD) and cognitive dysfunction (POCD). S100A12 is involved in inflammatory process and is recently known as a biomarker for brain injury. Herein, we clarified whether serum S100A12 levels are related to POD and POCD after hip fracture surgery in elderly patients.

**Materials and Methods:**

In this prospective, observational study, we gauged S100A12 levels in preoperative and postoperative serum from 186 patients and serum from 186 controls. Patients were categorized according to the presence of POD and POCD.

**Results:**

Postoperative, but not preoperative serum S100A12 levels were significantly higher in patients than in controls. There was a positive and independent correlation between postoperative C‐reactive protein and S100A12 levels (*t* = 8.797, *p* < 0.001). Postoperative S10012 levels and age were independently associated with the risk of developing POD (S100A12 levels: odds ratio [OR] = 1.166, 95% confidence interval [CI] = 1.045–2.087, *p* = 0.001; age: OR = 1.243, 95% CI = 1.073–1.419, *p* = 0.012) and POCD (S100A12: OR = 1.157, 95% CI = 1.030–1.986, *p* = 0.003; age: OR = 1.228, 95% CI = 1.054–1.387, *p* = 0.014). In terms of area under receiver operating characteristic curve, postoperative S100A12 levels had a higher predictive ability than age and their combination dramatically exceeded that of each one alone.

**Conclusions:**

Postoperative elevated serum S100A12 levels have a strong relation to inflammation and are associated independently with the development of POD and POCD, substantializing serum S100A12 as a potential biomarker for predicting POD and POCD in elderly patients undergoing hip fracture surgery.

## 1 INTRODUCTION

In elderly population undergoing hip fracture surgery, postoperative delirium (POD) and cognitive dysfunction (POCD) are the two frequent complications and also are the important causes of morbidity, mortality, and prolonged hospital stay (Edlund, Lundstrom, Lundstrom, Hedqvist, & Gustafson, 1999; Francis & Kapoor, 1992; Kratz, Heinrich, Schlauß, & Diefenbacher, 2015; Sanguineti, Wild, & Fain, 2014). There are many causes of POD and POCD, as well as the pathophysiology remains uncertain and possibly is multifactorial. However, some mechanisms have been proposed, among which inflammation and its related brain injury play the key roles (Cerejeira, Nogueira, Luís, Vaz‐Serra, & Mukaetova‐Ladinska, 2012; Neerland et al., 2016; Vasunilashorn et al., 2017). The best managements of POD and POCD are prevention through recognition of those patients who are at high risk. Treatment comprises both nonpharmacologic and pharmacologic interventions. Prognosis is excellent if recognized early and managed effectively (Lipowski, 1989; Lundstrom, Edlund, Bucht, Karlsson, & Gustafson, 2003; Nie, Zhao, Zhang, Jiang, & Yang, 2012). Therefore, early prediction for POD and POCD is very important for such patients.

S100A12 is a member of the S100 gene family of calcium‐binding proteins. It plays a crucial role in immune response and acts as a damage‐associated molecular pattern protein, signaling through the receptor for advanced glycation end products (RAGE) (Jung et al., 2017; Khorramdelazad et al., 2015; Zhao et al., 2016). Clearly, RAGE has been found in the central nervous system and emerges as a central regulator of brain inflammatory processes (Hu et al., 2018; Li et al., 2017; Villarreal et al., 2014). S100A12 expressions are up‐regulated significantly in brain tissues of patients with Alzheimer's disease (Shepherd et al., 2006). Recently, circulating S100A12 levels have been verified to be highly associated with inflammation, severity, mortality, and functional outcome in patients with acute brain injury, such as acute ischemic stroke, spontaneous intracerebral hemorrhage, and traumatic brain injury (Feng et al., 2018; Qian, He, Li, Qian, & Zheng, 2018; Wakisaka et al., 2014). Such data have shown that S100A12 acts as a pro‐inflammatory cytokine and becomes a biomarker for brain injury. However, circulating S100A12 levels are not investigated in patients with delirium or cognitive dysfunction. In this study, we recruited a group of elder patients needing hip fracture surgery, measured their preoperative and postoperative S100A12 levels and further assessed the relationship between S100A12 levels and development of POD and POCD.

## 2 MATERIALS AND METHODS

### 2.1 Study design, setting, and participants

We carried out a prospective, observational study in the Affiliated Hangzhou First People's Hospital, School of Medicine, Zhejiang University in Hangzhou, Zhejiang Province, China. The recruitment period was from May 2014 to May 2017. The recruited subjects were the elderly healthy individuals (defined as ≥age of 65 years) (as the controls) and the elderly patients undergoing surgery for a femoral neck fracture or an intertrochanteric fracture (as the cases). We excluded such patients with Mini‐Mental State Examination score <24 before surgery, previous psychiatric disorder like dementia, cognitive dysfunction, delirium, or depressive illness, any severe visual or auditory disorders, inability to speak or understand Chinese, infection within recent 1 month, prior or present neurological diseases like ischemic or hemorrhagic stroke and severe head trauma, uremia, liver cirrhosis, or malignancy. Criteria for inclusion as healthy control were absence of delirium, cognitive dysfunction, dementia, infection within recent 1 month, prior or present neurological diseases like ischemic or hemorrhagic stroke and severe head trauma, uremia, liver cirrhosis, and malignancy. All study participants gave written informed consent, and the study was approved by the institutional ethics committee for bio‐medical research at our hospital. This work has been done according to The Code of Ethics of the World Medical Association (Declaration of Helsinki).

### 2.2 Data sources and variables

The demographic data, including gender, age, and body mass index along with other relevant clinical data, were recorded. The data were obtained directly from patients or their relatives and healthy individuals during an interview performed at initial recruitment. We also recorded fracture type (femoral neck fracture or intertrochanteric fracture), surgical delay, type of anesthesia (spinal or general), duration of anesthesia, amount of transfusion, type of operation (hip arthroplasty or internal fixation), and hospitalization after surgery. Surgical risk was estimated according to the American Society of Anesthesiologists rating scale (Owens, Felts, & Spitznagel, 1978). Medical comorbidities were assessed based on the modified Charlson's Comorbidity Index (Charlson, Pompei, Ales, & MacKenzie, 1987). Patients were evaluated for delirium twice daily until the seventh postoperative day. Delirium was diagnosed according to the Confusion Assessment Method (Ely, Inouye, et al., 2001; Ely, Margolin, et al., 2001). Neuropsychological assessment was conducted 1 day before and one week after surgery. POCD was diagnosed in accordance with the method used in the ISPOCD1 study (Moller et al., 1998).

### 2.3 Measurements

Peripheral venous blood was drawn after overnight fasting from healthy controls at initial recruitment and from patients on the first preoperative and postoperative day. Fasting blood glucose was determined using the Accu‐Chek Performa (Roche Diagnostics GmbH, Mannheim‐Germany). For the determination of serum S100A12 and C‐reactive protein levels, samples were centrifuged at 3,000 g, aliquoted, and frozen at −70°C until assayed. Immune markers, including S100A12 and C‐reactive protein, were quantified in duplicates with commercially available enzyme‐linked immunosorbent assay kits (CUSABIO, Wuhan, China) following the manufacturer's protocol. The mean values of two measurements were utilized for analyses. All determinations were performed in batches every 3 months by the same laboratory technician blinded to all clinical data.

### 2.4 Statistical methods

The normality of data distribution was analyzed using Kolmogorov–Smirnov test or Shapiro–Wilk test. All continuous data were non‐normally distributed and thereby presented as median (interquartile range). All categorical data were reported as count (percentage). Statistical differences between two groups were investigated using the Mann–Whitney *U* test or Wilcoxon rank‐sum test for continuous data and the chi‐square test or Fisher exact test for categorical data. Multiple comparisons were adjusted using Bonferroni test. Bivariate correlations were carried out using Spearman's correlation coefficient. Those variables verified to be significantly associated were further incorporated into multivariate linear regression to analyze the association of S100A12 levels with C‐reactive protein levels. Binary logistic regression was conducted for adjustments for potential confounders and for assessing predictors of POD and POCD. Univariate logistic regression models were configured, and those variables found to be predictors of POD and POCD were then analyzed with multivariate model. The odds ratio (OR) values and 95% confidence intervals (CIs) were estimated. Receiver operating characteristic (ROC) curves were configured to determine cutoff values for optimal predictive sensitivities and specificities. The area under curves (AUCs) and 95% CI were calculated. A combined logistic regression model was used to assess the additive benefit of S100A12 levels to age. SPSS 19.0 (SPSS Inc., Chicago, IL, USA) was used for data analysis, and values of *p* < 0.05 were considered significant. Graphics were done with GraphPad Prism Software version 5.00 for Windows (GraphPad Software, San Diego California, USA). ROC analysis was performed using MedCalc 9.6.4.0 (MedCalc Software, Mariakerke, Belgium).

## 3 RESULTS

### 3.1 Study population characteristics

During recruitment period, we firstly enrolled a total of 238 elderly patients undergoing surgery for a femoral neck fracture or an intertrochanteric fracture. According to the exclusion criteria, we excluded 6 patients with preoperative Mini‐Mental State Examination score <24, 9 patients with previous psychiatric disorder like dementia, cognitive dysfunction, delirium, or depressive illness, 5 patients with any severe visual or auditory disorders, 5 patients unable to speak or understand Chinese, 8 patients suffering from infection within recent 1 month, 9 patients having prior or present neurological diseases like ischemic or hemorrhagic stroke and severe head trauma as well as 10 patients with other severe diseases, such as uremia, liver cirrhosis, and malignancy. Ultimately, one hundred eighty‐six patients were assessed. Meantime, we recruited 186 elderly controls that had similar age, body mass index, and percentage of gender as compared to the patients. The clinical and laboratory characteristics of the patients are listed in Table 1.

**Table 1:** Clinical and laboratory characteristics and parameters correlated with serum S100A12 levels after hip fracture surgery in elderly patients

Variable	Range	Number/Median (IQR)	Correlation analysis	
			*r* value	*p* value
Gender (male/female)		78/108	0.117	0.113
Age (year)	65–91	73 (68–81)	0.143	0.052
Body mass index (kg/m^2^)	18.5–33.1	23.1 (21.4–25.3)	0.120	0.102
Type of fracture (femoral neck fracture/intertrochanteric fracture)		98/88	0.101	0.170
Modified Charlson's Comorbidity Index	0–4	1 (1–2)	0.174	0.017
American Society of Anesthesiologists Scale (Ⅰ/Ⅱ/Ⅲ)		14/104/68	0.163	0.026
Type of anesthesia (spinal/general)		102/84	0.103	0.163
Duration of anesthesia (min)	68–181	101 (92–109)	0.063	0.394
Amount of transfusion (ml)	0–500	300 (200–400)	0.075	0.312
Delay of surgery (days)	1–18	6 (2–10)	0.113	0.124
Hospitalization after surgery (days)	10–28	14 (12–16)	0.118	0.108
Type of surgery (internal fixation/arthroplasty)		37/149	0.092	0.213
Blood glucose levels (mmol/L)	2.9–22.6	7.3 (5.2–10.6)	0.268	<0.001
Serum C‐reactive protein levels (mg/L)	2.2–29.4	12.4 (7.1–15.1)	0.559	<0.001

Notes:

The continuous data were presented as median (interquartile range [IQR]), and the categorical data were reported as count. Bivariate correlation was assessed using Spearman's correlation coefficient.

### 3.2 Serum S100A12 levels and other variables

**Figure 1:**
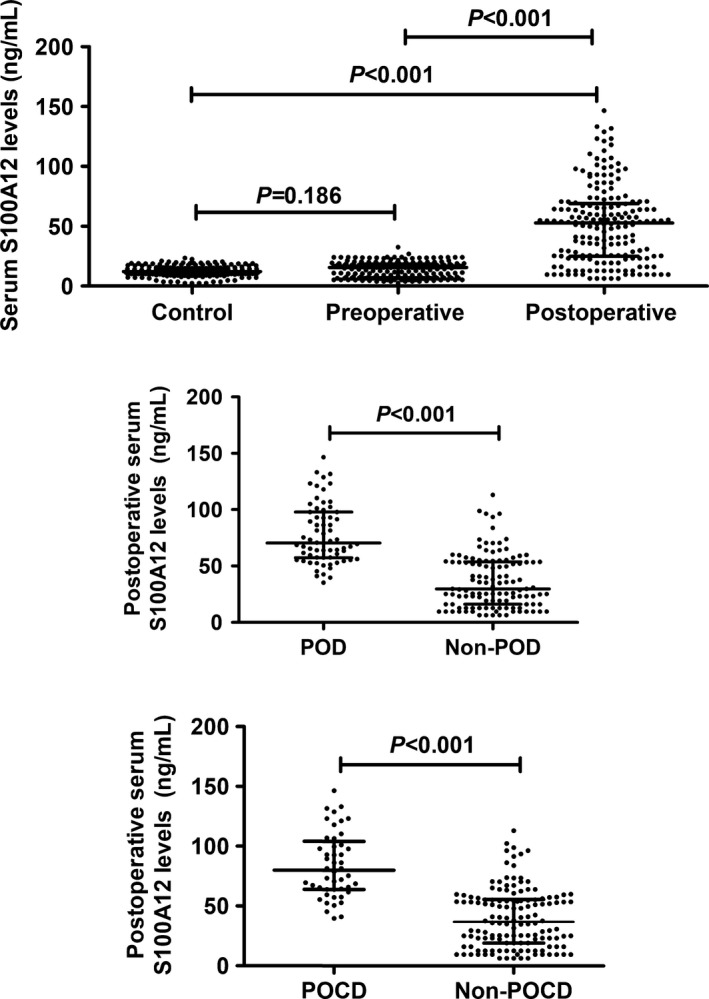
Comparisons of serum S100A12 levels between the controls and the patients, between the patients with postoperative delirium (POD) and those without POD, as well as between the patients with postoperative cognitive dysfunction (POCD) and those without POCD. This figure shows that there was not statistically significant difference between preoperative serum S100A12 levels of patients and serum S100A12 levels of controls; as compared with preoperative S100A12 levels in patients, postoperative S100A12 levels were significantly elevated in patients; consistently, postoperative S100A12 levels in patients were significantly higher than those in controls; alternatively, serum S100A12 levels were significantly higher in POD patients than in patients without POD as well as in POCD patients than in non‐POCD patients

In Figure 1, there was not statistically significant difference between preoperative serum S100A12 levels of patients and serum S100A12 levels of controls; as compared with preoperative S100A12 levels in patients, postoperative S100A12 levels were significantly elevated in patients; consistently, postoperative S100A12 levels in patients were significantly higher than those in controls. After correction by Bonferroni test, the differences still remained significant. In addition, a total of 67 patients (36.0%) experienced POD and 48 patients (25.8%) had POCD; serum S100A12 levels were significantly higher in POD patients than in patients without POD as well as in POCD patients compared to non‐POCD patients.

**Figure 2:**
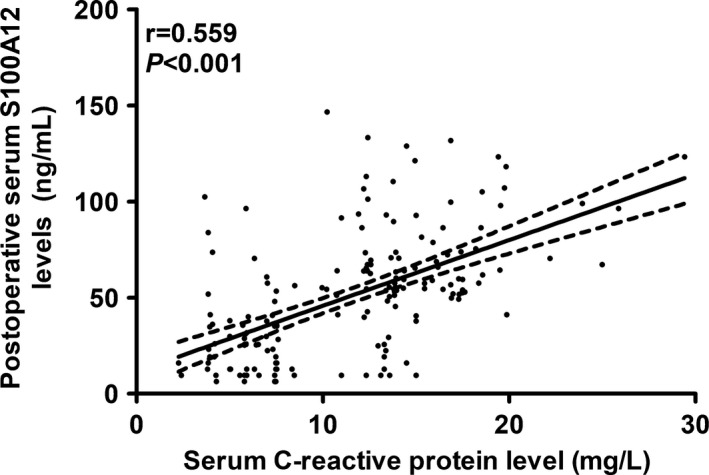
Relationship between postoperative serum S100A12 levels and serum C‐reactive protein levels in elderly patients undergoing hip fracture surgery. Just as depicted in this Figure, postoperative serum S100A12 levels were significantly correlated with serum C‐reactive protein levels

In Table 1, bivariate correlation analysis showed that postoperative serum S100A12 levels were significantly correlated with modified Charlson's Comorbidity Index, American Society of Anesthesiologists Scale, blood glucose levels, and serum C‐reactive protein levels. We incorporated the preceding significant variables into a multivariate linear regression model and subsequently found that serum S100A12 levels were independently associated with serum C‐reactive protein levels (*t* = 8.797, *p* < 0.001; Figure 2).

### 3.3 POD prediction

In Table 2, we used univariate analysis and thereby demonstrated that the POD patients had older age, higher modified Charlson's Comorbidity Index, higher preoperative American Society of Anesthesiologists grade, higher postoperative serum S100A12 levels, higher serum C‐reactive protein levels, higher blood glucose levels, longer duration of anesthesia, and more prolonged hospitalization after surgery; a higher percentage of POD patients underwent general anesthesia and arthroplasty. Furthermore, the above‐mentioned parameters verified to be significant in univariate analysis were incorporated into the binary logistic regression model, and subsequently, it was revealed that S100A12 levels (OR = 1.166, 95% CI = 1.045–2.087, *p* = 0.001) and age (OR = 1.243, 95% CI = 1.073–1.419, *p* = 0.012) were the two independent predictors for POD.

**Table 2:** The factors related to delirium after hip fracture surgery in elderly patients

Variable	PD	Non‐PD	*p* value	OR (95% CI)	*p* value
Gender (male/female)	29/38	49/70	0.780	1.090 (0.595–1.998)	0.781
Age (year)	77 (74–84)	71 (68–80)	<0.001	1.130 (1.077–1.186)	<0.001
Body mass index (kg/m^2^)	23.4 (21.6–26.6)	23.1 (21.4–25.1)	0.329	1.052 (0.936–1.182)	0.393
Type of fracture (femoral neck fracture/intertrochanteric fracture)	40/27	58/61	0.151	1.213 (0.627–2.349)	0.566
Modified Charlson's Comorbidity Index	1 (1–2)	1 (0–2)	0.039	1.389 (1.040–1.856)	0.026
American Society of Anesthesiologists Scale (Ⅰ/Ⅱ/Ⅲ)	4/29/34	10/75/34	0.011	2.057 (1.209–3.499)	0.008
Type of anesthesia (spinal/general)	26/41	76/43	0.001	2.787 (1.503–5.168)	0.001
Duration of anesthesia (min)	104 (93–115)	99 (91–106)	0.009	1.027 (1.009–1.45)	0.003
Amount of transfusion (ml)	320 (220–410)	300 (200–420)	0.794	1.001 (0.997–1.005)	0.662
Delay of surgery (days)	7 (2–10)	6 (2–11)	0.387	1.012 (0.956–1.070)	0.691
Hospitalization after surgery (days)	16 (12–18)	13 (12–15)	0.019	1.117 (1.023–1.218)	0.013
Type of surgery (internal fixation/arthroplasty)	7/60	30/89	0.015	2.889 (1.192–7.004)	0.019
Blood glucose levels (mmol/L)	8.4 (6.6–11.5)	5.8 (4.0–10.4)	<0.001	1.142 (1.054–1.237)	0.001
Serum C‐reactive protein levels (mg/L)	13.9 (12.4–17.5)	7.5 (5.8–13.8)	<0.001	1.239 (1.147–1.337)	<0.001
Serum S100A12 levels (ng/ml)	70.4 (57.4–97.9)	29.8 (16.0–53.8)	<0.001	1.066 (1.046–1.086)	<0.001

Notes:

The categorical and continuous variables were expressed as counts (percentages) and median (interquartile range), respectively. Intergroup comparisons were done using the chi‐square test or Fisher exact test for categorical variables and the Mann–Whitney *U* test for continuous variables. The predictors of postoperative delirium (POD) were identified using a univariate logistic regression analysis. The odds ratio (OR) values and 95% confidence intervals (CIs) were presented.

**Figure 3:**
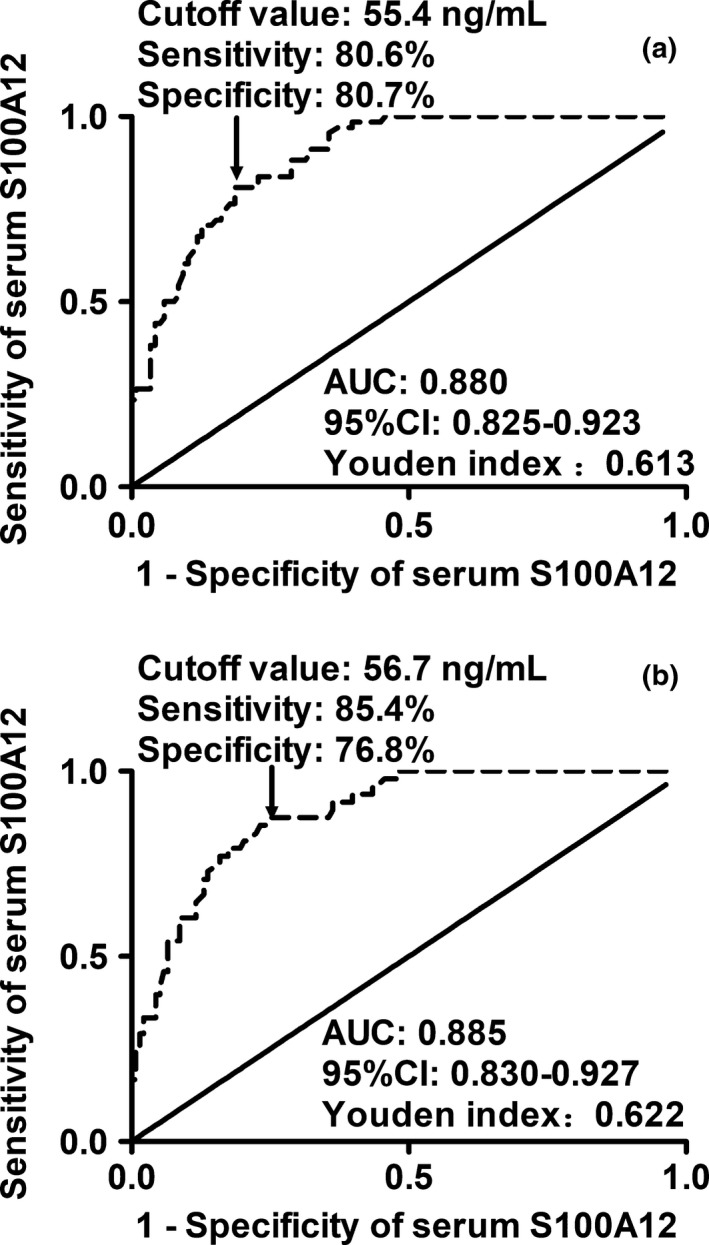
Receiver operating characteristic curve analysis of postoperative serum S100A12 levels for discriminating postoperative delirium (a) and postoperative cognitive dysfunction (b) in elderly patients undergoing hip fracture surgery. Just as portrayed in this Figure, an optimal value of serum S100A12 levels determined postoperatively was selected, which distinguished patients developing postoperative delirium and postoperative cognitive dysfunction with the corresponding sensitivity and specificity values

In Figure 3, under the ROC curve, an optimal cutoff value of S100A12 levels (55.4 ng/ml) was chosen, which discriminated patients developing POD with 80.6% sensitivity and 80.7% specificity values. Its mean AUC was 0.880 (95% CI, 0.825–0.923). In terms of the AUC, the discriminatory ability of S100A12 levels was significantly higher than that of age (AUC 0.740, 95% CI 0.671–0.801, *p* = 0.001). Using a combined logistic regression model, we found that serum S100A12 combined with age (combined AUC 0.912, 95% CI 0.861–0.948) significantly improved AUCs of both age and serum S100A12 alone (*p* < 0.001 and *p* = 0.022, respectively).

### 3.4 POCD prediction

Just as listed in Table 3, the univariate analysis demonstrated that older age, higher modified Charlson's Comorbidity Index, higher preoperative American Society of Anesthesiologists grade, higher postoperative serum S100A12 levels, higher serum C‐reactive protein levels, higher blood glucose levels, longer duration of anesthesia, more prolonged hospitalization after surgery, and a higher percentage of general anesthesia and arthroplasty were significantly associated with increasing risk of developing POCD. When those aforementioned significant parameters were further analyzed in the multivariate logistic model, serum S100A12 and age independently discriminated the development of POCD with OR values of 1.157 (95% CI = 1.030–1.986, *p* = 0.003) and 1.228 (95% CI = 1.054–1.387, *p* = 0.014), respectively.

**Table 3:** The factors related to cognitive dysfunction after hip fracture surgery in elderly patients

Variable	POCD	Non‐POCD	*p* value	OR (95% CI)	*p* value
Gender (male/female)	21/27	57/81	0.767	1.105 (0.569–2.146)	0.769
Age (year)	79 (74–85)	71 (68–80)	<0.001	1.118 (1.063–1.177)	<0.001
Body mass index (kg/m^2^)	23.6 (21.3–25.6)	23.1 (21.7–25.1)	0.741	1.023 (0.901–1.162)	0.723
Type of fracture (femoral neck fracture/intertrochanteric fracture)	27/21	71/67	0.566	1.558 (0.850–2.857)	0.152
Modified Charlson's Comorbidity Index	1 (1–2)	1 (0–2)	0.017	1.585 (1.159–2.167)	0.014
American Society of Anesthesiologists Scale (Ⅰ/Ⅱ/Ⅲ)	2/19/27	12/85/41	0.004	2.552 (1.397–4.663)	0.002
Type of anesthesia (spinal/general)	18/30	84/54	0.005	2.593 (1.317–5.102)	0.006
Duration of anesthesia (min)	105 (94–115)	99 (89–108)	0.006	1.031 (1.012–1.050)	0.001
Amount of transfusion (ml)	310 (210–400)	305 (215–420)	0.794	1.002 (0.996–1.008)	0.897
Delay of surgery (days)	8 (2–9)	6 (2–11)	0.236	1.019 (0.958–1.084)	0.549
Hospitalization after surgery (days)	17 (11–19)	13 (11–15)	0.010	1.134 (1.035–1.244)	0.007
Type of surgery (internal fixation/arthroplasty)	3/45	34/104	0.006	4.904 (1.432–16.797)	0.011
Blood glucose levels (mmol/L)	8.5 (6.7–12.0)	6.1 (4.1–10.4)	<0.001	1.139 (1.047–1.240)	0.003
Serum C‐reactive protein levels (mg/L)	15.2 (13.4–17.6)	8.4 (5.9–13.9)	<0.001	1.275 (1.165–1.396)	<0.001
Serum S100A12 levels (ng/ml)	80.1 (63.9–104.1)	37.0 (19.2–55.6)	<0.001	1.063 (1.043–1.083)	<0.001

Notes:

The categorical and continuous variables were expressed as counts (percentages) and median (interquartile range), respectively. Intergroup comparisons were done using the chi‐square test or Fisher exact test for categorical variables and the Mann–Whitney *U* test for continuous variables. The predictors of postoperative cognitive dysfunction (POCD) were identified using a univariate logistic regression analysis. The odds ratio (OR) values and 95% confidence intervals (CIs) were presented.

Just as depicted in Figure 3, a ROC curve selected a suitable cutoff value of serum S100A12 level (56.7 ng/ml) as an indicator for predicting POCD, which generated a sensitivity value of 85.4% and a specificity value of 76.8%. As compared with age (AUC 0.721, 95% CI 0.651–0.784), serum S100A12 levels had significantly higher discriminatory ability for differentiating patients at risk of POCD (*p* < 0.001). Moreover, the predictive performance of combination of serum S100A12 and age (combined AUC 0.926, 95% CI 0.879–0.959) significantly exceeded those of both age and serum S100A12 alone (*p* < 0.001 and *p* = 0.007, respectively) in a combined logistic regression model.

## 4 DISCUSSION

To the best of our knowledge, this prospective, observational study, for the first time, measured S100A12 levels in the peripheral blood derived from patients with the development of POD or POCD. We collected clinical and laboratory data,as well as found some interesting results as follows: (a) postoperative, but not preoperative serum S100A12 levels in all patients were significantly higher than in the controls; (b) postoperative serum S100A12 levels were obviously higher in patients with the development of POD or POCD than in other remaining patients; (c) age and serum S100A12 emerged as the two independent predictors for POD and POCD; (d) in accordance with AUC, serum S100A12 levels had higher discriminatory ability for patients at risk of POD or POCD than age; (e) S100A12 levels markedly enhanced the predictive performance of age for POD or POCD; and (f) serum S100A12 levels were independently related to serum C‐reactive protein levels. Hence, those data implied that S100A12 might be implicated in inflammatory process during the development of POD and POCD in elderly patients needing hip fracture operation and serum S100A12 should be a potential biomarker for discriminating the elderly patients developing POD or POCD following hip fracture surgery.

Although other variables are reported to be associated with risk of POD and POCD, age is always a well‐established predictor for the occurrences of both POD and POCD (Afonso et al., 2010; Bruce, Ritchie, Blizard, Lai, & Raven, 2007; Jonghe et al., 2007; Koster, Oosterveld, Hensens, Wijma, & Palen, 2008; Tan et al., 2008). Similarly, our study also found that a higher percentage of POD and POCD happened in older elderly patients undergoing hip fracture surgery. Moreover, undoubtedly, age emerged as an independent risk factor for the occurrences of both POD and POCD. There are very complex mechanisms involved in pathogenesis of POD and POCD. And, it is unclear why elderly patients are apt to the development of POD and POCD. Some explanations have been mentioned. Firstly, with increasing age, incidence of atherosclerosis and endothelial dysfunction is obviously elevated (Rudolph et al., 2009), and therefore, elderly patients are more easily at risk of cerebral embolization. Furthermore, cerebral atherosclerosis combined with postoperative inflammatory and oxidative changes may decrease cerebral blood flow (Rudolph et al., 2008). There are also neurochemical factors making elderly patients to easily suffer from POD and POCD, such as lack of cholinergic reserves (Rudolph et al., 2008). In summary, age might be a potential predictor for the development of POD and POCD after hip fracture surgery in elderly patients. However, we configure a ROC curve and subsequently found that age had a medium discriminatory ability for patients at risk of POD and POCD. As a consequence, another biomarker should be explored to supplement age for predicting the occurrences of POD and POCD.

Although the exact etiology and pathogenesis of POD and POCD remain unclear, it is generally accepted that their occurrences are closely related to cerebral neuronal damage which is caused by imbalance of noradrenergic/cholinergic neurotransmission, perioperative hypoxia, micro‐embolisms, or hypotension (Herrmann, Ebert, Tober, Hann, & Huth, 1999; Munster et al., 2009; Rasmussen, Christiansen, Rasmussen, Kristensen, & Moller, 2000). Furthermore, it is well known that inflammatory mediators play a pivotal role in driving activation of glial cell that leads to inflammation and brain injury during POD and POCD (Cerejeira et al., 2012; Neerland et al., 2016; Vasunilashorn et al., 2017). Accumulating evidence has disclosed high levels of several inflammatory mediators including C‐reactive protein, which are present at very high levels in the peripheral blood of patients at risk of POD or POCD (Cerejeira et al., 2012; Neerland et al., 2016; Vasunilashorn et al., 2017). It is confirmed initially that human S100A12 is expressed and secreted by activated neutrophil granulocytes (Achouiti et al., 2013; Bae et al., 2014; He et al., 2015; Isoyama et al., 2015, 2016; Zhao et al., 2013). In this regard, S100A12 is known to trigger a pro‐inflammatory immune response by binding RAGE, thereby leading to inflammatory activation (Achouiti et al., 2013; Bae et al., 2014; He et al., 2015; Isoyama et al., 2015, 2016; Zhao et al., 2013). Actually, S100A12 can be secreted from glial cells and its expression is obviously enhanced under some pathological conditions, like Alzheimer's disease (Shepherd et al., 2006). A previous clinical study has demonstrated that circulating S100A12 levels were correlated with C‐reactive protein level in peripheral blood derived from patients with severe traumatic brain injury (Feng et al., 2018), indicating that S100A12 might be involved in inflammation after brain injury. With the difference to other study in statistical method (Feng et al., 2018), we used multivariate linear regression model and also found the similar result that serum C‐reactive protein levels were independently associated with postoperative S100A12 levels in serum obtained from the elderly patients with hip fracture surgery. The accumulating evidence implies that S100A12 might be involved in inflammation underlying pathophysiological process of POD and POCD.

Some previous studies have confirmed that S100A12 levels in the peripheral blood reflected the severity and poor prognosis of patients with ischemic stroke, spontaneous intracerebral hemorrhage and severe traumatic brain injury (Feng et al., 2018; Qian et al., 2018; Wakisaka et al., 2014). The preceding studies have proposed that S100A12 should have the potential to be a good biomarker for reflecting the extent of brain injury. The current study was designed to elucidate the relationships between serum S100A12 levels and the development of POD and POCD in the elder patients undergoing hip fracture surgery. Univariate analysis showed that patients at risk of POD or POCD had higher postoperative serum S100A12 levels than those without the development of POD or POCD. Furthermore, we configured multivariate logistic regression models to verify independent association of postoperative serum S100A12 levels with the development of POD and POCD. Intriguingly, serum S100A12 and age were identified as the two independent predictors for POD and POCD development. Importantly, its discriminative ability significantly exceeded that of age in terms of AUC. Astonishingly, in a combined logistic regression model, serum S100A12 combined with age statistically significantly enhanced the discriminatory ability of both age and serum S100A12 alone for predicting development of POD and POCD. Based on our data, we deduced that postoperative determination of serum S100A12 levels could be helpful to distinguish elder patients developing POD or POCD after hip fracture surgery.

## 5 CONCLUSIONS

Our current study shows that postoperative elevation of serum S100A12 levels is independently correlated with serum C‐reactive protein levels as well as associated with development of POD and POCD; serum S100A12, than age, possesses a higher predictive value for patients at risk of POD and POCD after hip fracture surgery in elder patients. Taken together, S100A12 might be involved in inflammation during development of POD and POCD, as well as its clinical determination might be helpful to differentiate elder patients at risk of POD and POCD after hip fracture surgery.

## DISCLOSURE

The authors declare that they have no competing interests.

## CONFLICT OF INTEREST

The authors have no conflict of interest.
